# Anakinra treatment for refractory cerebral autoinflammatory responses

**DOI:** 10.1002/acn3.51500

**Published:** 2022-01-18

**Authors:** Yoonhyuk Jang, Woo‐Jin Lee, Han Sang Lee, Kon Chu, Sang Kun Lee, Soon‐Tae Lee

**Affiliations:** ^1^ Department of Neurology Seoul National University Hospital Seoul South Korea

## Abstract

Refractory cerebral autoinflammatory–autoimmune diseases are often associated with dysregulated innate immunity and are targeted by anakinra, an interleukin‐1 receptor antagonist. We analyzed the therapeutic effect of anakinra in refractory cerebral autoinflammatory response (CAIR) at a single institution from January 2017 to May 2021. In total, 12 patients with various etiologies were sympathetically treated with anakinra (100 mg/day subcutaneously). Four patients showed good responses, and among these patients, three patients had pathologically demonstrated CAIR. The other eight patients were nonresponsive. No patient had a serious adverse effect. Thus, anakinra may be a therapeutic option for refractory cerebral autoinflammatory diseases.

## Introduction

Cerebral autoinflammatory–autoimmune diseases, including autoimmune encephalitis, multiple sclerosis (MS), acute disseminating encephalomyelitis (ADEM), cryptogenic new‐onset refractory status epilepticus (NORSE), and other miscellaneous diseases, are often refractory to conventional immunosuppressive treatments, such as intravenous immunoglobulin (IVIg), plasmapheresis, rituximab, and tocilizumab, which target adaptive immune‐mediated responses, such as B and T cells.^1^, ^2^, ^3^, ^4^, ^5^ However, accumulating evidence suggests that dysregulated innate immunity mediated by microglia, macrophages, and interleukin‐1 (IL‐1) plays key roles in refractoriness,^6^, ^7^, ^8^ and this pathological situation could be defined as the cerebral autoinflammatory response (CAIR) under the following conditions: (1) pathological evidence of increased cerebral inflammation mediated by innate immune cells, (2) minimal engagement of autoantibodies or autoreactive T cells, and (3) exclusion of alternative causes.^7^

Anakinra is an interleukin‐1 receptor (IL‐1R) antagonist that has shown efficacy and safety in refractory rheumatoid diseases^9^, ^10^ by inhibiting the innate immune response mediated by IL‐1, macrophages, and proinflammatory cascades.^11^ Anakinra has a low molecular weight, has the potential for blood–brain barrier penetrance^12^ and has shown possible therapeutic potential in cases with febrile infection‐related epilepsy syndrome and CAIR.^7^, ^13^ After selecting patients with CAIR by brain biopsy or clinical implication, anakinra might be used to treat the unbalanced innate immunity pathogenesis in monotherapy or in combination with adaptive immunotherapeutic drugs.

Here, we aimed to analyze the therapeutic potential of anakinra in patients with CAIR evident in brain biopsy or theoretically. The patients were from a single institutional cohort that included all consecutive patients who received therapy for a sympathetic purpose for intractable cerebral autoinflammatory–autoimmune diseases.

## Methods

From a single institutional cohort, we selected patients who received anakinra treatment from January 2017 to May 2021. Anakinra is sympathetically used in patients with intractable central nervous system diseases caused by autoimmune, autoinflammatory, postviral, or other miscellaneous caused and evidence of innate immunity pathogenesis based on brain biopsies or clinical implications. In the patients, CAIR was demonstrated by pathology of macrophage infiltration greater than a moderate degree. Moreover, patients with refractory cerebral autoinflammatory–autoimmune diseases, such as seronegative autoimmune encephalitis, MS, ADEM, neurotoxic encephalitis, and cryptogenic NORSE, who were unresponsive to previous immunotherapies (steroid, IVIg, rituximab, tocilizumab, tofacitinib, and other conventional immunosuppressant drugs) were also included.

Anakinra was administered daily by subcutaneous injection at 100 mg per day. The treating physicians decided the duration of therapy based on the clinical response. The outcome was evaluated with the modified Rankin Scale (mRS), Clinical Assessment Scale in Autoimmune Encephalitis (CASE), and Expanded Disability Status Scale (EDSS) for MS.^14^ A good response to anakinra treatment was defined as any improvement in mRS, CASE, and EDSS or seizure cessation. A very good response was defined as an improvement in unassisted daily activity or a mRS score of 2.

## Results

### Characteristics and treatment response of patients

In total, 12 patients were treated with anakinra (Table 1). The clinical diagnoses were primary progressive multiple sclerosis (PPMS) (Patient 1), granulomatosis with polyangiitis (GPA) (Patient 2), ADEM (Patients 3, 4, and 5), NORSE (Patients 6 and 7), seronegative autoimmune encephalitis (Patient 8), autoimmune meningitis (Patient 9), methotrexate‐necrotizing leukoencephalopathy (Patient 10), Japanese B encephalitis (JBE) (Patient 11), and sporadic Creutzfledt–Jakob disease (CJD) (Patient 12). Brain biopsy was performed in seven patients (Patients 1, 2, 3, 5, 8, and 12), and three patients (Patients 1, 2, and 3) showed CAIR as defined by prominent CD68‐positive macrophage/microglial infiltration. All patients were refractory to previous immunotherapies (Table 1).

**Table 1:** Characteristics and treatment response of patients treated with anakinra.

Patient ID	1	2	3	4	5	6	7	8	9	10	11	12
Age/sex	29/F	51/M	54/M	24/M	56/M	19/F	18/F	50/F	74/M	45/F	56/M	65/M
Clinical symptoms/signs	Gait disturbance, urinary retention, fecal inconsistency	Fever, altered mentality, memory impairment, seizure	Fever, altered mentality	Fever, Altered mentality, seizure, weakness in both legs, gaze palsy, hiccups, dysphagia	General weakness, altered mentality, memory impairment, Dysarthria	Fever, seizure	Fever, seizure	Depression, cognitive decline, dysarthria, dysphagia, gait disturbance, seizure	Fever, cognitive decline, memory impairment, gait disturbance	Altered mentality	Fever, altered mentality	Anxiety, cognitive decline, memory impairment, seizure, gait disturbance
Diagnosis	CAIR with PPMS	CAIR with GPA	CAIR with ADEM	ADEM	ADEM, chronic stage	NORSE	NORSE	Seronegative AE	Autoimmune meningitis	MTX‐necrotizing leukoencephalopathy	JBE	Sporadic CJD
Brain biopsy	CD68 (++): Moderate microglia infiltration	CD68 (+++): Diffuse microglia infiltration with chronic granulomatous and suppurative vasculitis	CD68 (++): Moderate microglia infiltration	N/A	LFB (−): Multifocal demyelination around vein	N/A	N/A	CD68 (+): Mild nonfoamy microglia with dominant CD8+ T cell infiltration	CD68 (+): mild nonfoamy microglia	N/A	N/A	RT‐QuIC: PrP^sc^ (+)
CSF profile at worst	W8 (L8), Ptn47, IgG index 3.04	W855 (P565, L145, O145), Ptn229, IgG index 0.87	W2 (L2), Ptn237, IgG index 0.63	W150 (P4, L146, O0), Ptn272	W26 (P1, L25), Ptn 98	W2 (L2), Ptn25, IgG index 0.41	W2 (L2), Ptn 58	W16 (P2, L9, O5), Ptn 97	W240 (P158, L5, O77), Ptn 703, IgG index 0.64	N/A	W389 (L389), Ptn 92	W0, Ptn 90, IgG index 0.53
MRI lesions	T2 lesion in bilat. Subcortical white matter, periventricular white matter, middle cerebellar peduncle, and cerebellum	T2 lesion in bilat. Medial temporal lobes and basal ganglia with leptomeningeal enhancement	Diffusion restriction and T2 lesion in bilat. Subcortical white matter with enhancement	Diffusion restriction and T2 lesion in bilat. Basal ganglia, periventricular white matter, midbrain, pons, and cerebellum with leptomeningeal enhancement	T1 high signal intensity in Rt. subcortical area, T2 lesion at bilat. Cerebral white matter	T2 lesion in bilat. Basal frontal lobes, parietotemporal lobes, insular cortex, medial occipital lobes, and posteromedial thalami.	Diffusion restriction and T2 lesion in bilat. Caudate nucleus and putamen.	T2 lesion in bilat. Parasagittal white matter, temporal area, and insula	Diffusion restriction at Rt. parahippocampal gyrus and corpus callosum. T2 lesion in bilat. Cerebral white matter and mesial temporal lobes with leptomeningeal enhancement	Diffusion restriction and T2 lesion in bilat. Fronto‐temporal lobes and middle cerebellar peduncle with multifocal enhancement	T2 lesion in bilat. Basal ganglia, thalami, and brainstem with diffuse enhancement	Diffusion restriction at pancortical regions
ImmunoTx before anakinra	Steroid, IVIg, RTX, TCZ, Tofacitinib	Steroid, IVIg, RTX, TCZ	Steroid, IVIg, RTX, TCZ	Steroid, IVIg	Steroid, IVIg, CTX, MMF	Steroid, IVIg, RTX, TCZ, Tofacitinib	Steroid, IVIg, RTX, TCZ	Steroid, IVIg, MMF, RTX, TCZ	Steroid, IVIg, RTX, TCZ, proleukin	Steroid	IVIg	IVIg (before diagnosis)
Duration of disease onset to anakinra	4.5 years	10 weeks	4 weeks	7 weeks	8 months	4 weeks	5 weeks	14 months	12 months	2 months	11 months	5 months
Response to anakinra	Very good	Very good	Good	Good	Unclear	Unclear	Unclear	Unclear	Unclear	Unclear	Unclear	Unclear
Duration of anakinra	5 months	14 days	4 weeks	1 week	1 week	6 weeks	4 days	2 weeks	1 week	2 weeks	1 week	1 week
mRS change by anakinra	4➔2	5➔1	5➔4	5➔4	5➔5	5➔5	5➔5	5➔5	4➔4	5➔ 6	5➔5	5➔5
CASE change by anakinra	3➔1	12➔0	19➔8	9➔2	16➔16	20➔20	23➔23	18➔18	8➔9	17➔20	19➔19	17➔17
Adverse events	None	None	None	None	None	Neutropenia	None	None	Confusion	None	None	None

AE, autoimmune encephalitis; ADEM, acute disseminated encephalomyelitis; Bilat., bilateral; CASE, clinical assessment scale in autoimmune encephalitis; CAIR, cerebral autoinflammatory response; CJD, Creutzfeldt–Jakob disease; CSF, cerebrospinal fluid; F, female; GPA, granulomatosis with polyangiitis; IgG, immunoglobulin G; ImmunoTx, immunotherapy; IVIg, intravenous immunoglobulin; JBE, Japanese B encephalitis; L, lymphocytic cells; LFB, Luxol fast blue; M, male; MMF, mycophenolate mofetil; MRI, magnetic resonance imaging; mRS, modified Rankin Scale; MTX, methotrexate; N/A, not available; NORSE, new‐onset refractory status epilepticus; O, other cells; P, polymorphonuclear cells; PPMS, primary progressive multiple sclerosis; PrP^sc^, scrapie isoform of the prion protein; Ptn, protein; Rt., right; RT‐QuIC, the real‐time quaking‐induced conversion; RTX, rituximab; TCZ, tocilizumab.

**Figure 1:**
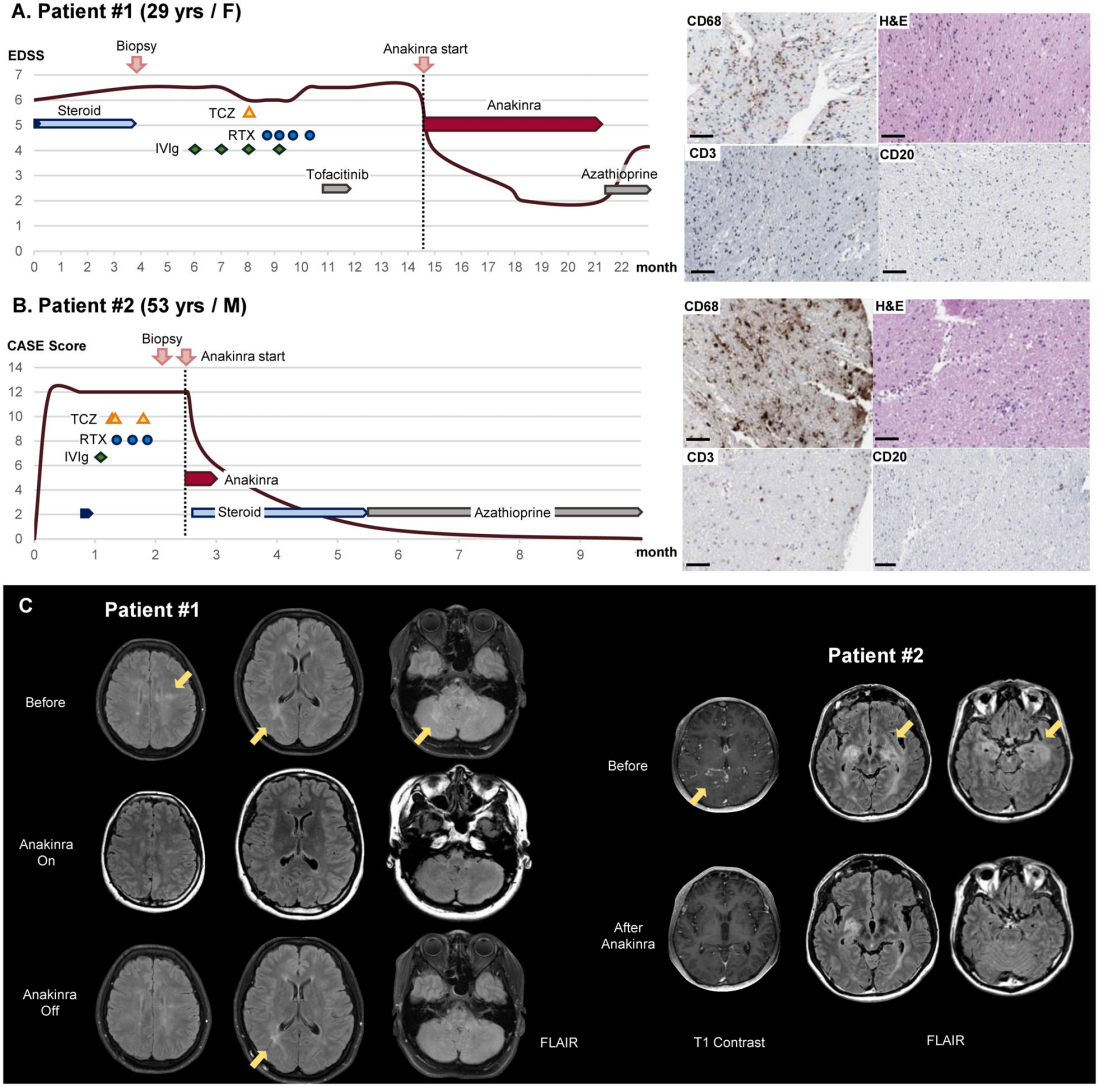
Clinical course, pathology, and brain images of Patients 1 and 2 who showed very good responses to the anakinra treatment. (A) A 29‐year‐old woman with primary progressive multiple sclerosis (PPMS) was pathologically identified as having a cerebral autoinflammatory response (CAIR), which is CD68‐positive macrophage infiltration. Anakinra treatment dramatically improved her symptoms from an expanded disability status scale (EDSS) score of 6.5 to 2.0, but after the discontinuation of anakinra, her symptoms aggravated again to an EDSS score of 4. (B) A 51‐year‐old man with refractory granulomatosis with polyangiitis (GPA) showed CD68‐positive microglia‐dominant pathology on brain biopsy. He responded very well to a 14‐day short‐term anakinra treatment followed by steroids and azathioprine, recovering from a Clinical Assessment Scale in Encephalitis (CASE) score of 12 to 0. All immunotherapies administered during the clinical courses are presented schematically. IVIg, intravenous immunoglobulin; RTX, rituximab; TCZ, tocilizumab. Scale bar = 100 μm. (C). In Patient 1, brain magnetic resonance imaging (MRI) identified T2 hyperintensity lesions in the bilateral subcortical and periventricular white matter, middle cerebellar peduncle, and cerebellum. During the anakinra treatment, the lesions disappeared but partially recurred after the discontinuation of anakinra. In Patient 2, brain MRI revealed improvement in T2 lesions in the bilateral medial temporal lobes and basal ganglia with leptomeningeal enhancement after the anakinra treatment.

The anakinra treatment elicited clear responses in four patients (Patients 1, 2, 3, and 4). In particular, all patients with biopsy‐proven CAIR (Patients 1, 2, and 3) had favorable outcomes following the anakinra treatment. Patient 1, who had PPMS, showed dramatic improvement in gait and magnetic resonance imaging (MRI) lesions during the anakinra treatment, but the disease recurred after switching from anakinra to azathioprine (Fig. 1A and C). Patient 2 was diagnosed with GPA and was unresponsive to previous immunotherapies, but anakinra had great efficacy in improving the clinical symptoms and MRI lesions (Fig. 1B and C). Patients 3 and 4, who were diagnosed with ADEM, also showed good responses to anakinra, and the clinical course and pathology of patient 3 have been previously described as a case report.^7^ However, the other eight patients (Patients 5, 6, 7, 8, 9, 10, 11, and 12) showed unclear responses to the anakinra treatment. Representative cases with very good responses to anakinra are described below.

Adverse events were reported in two patients (Patients 6 and 9). Patient 6 experienced mild neutropenia without fever. Patient 9 showed delirium during the anakinra treatment, resulting in treatment discontinuation.

### Case 1 (Patient 1)

A 29‐year‐old female was referred to our clinic due to progressive gait disability (Fig. 1A and C). She was previously healthy, but after delivery, she complained of gait disturbance with a tingling sensation in both legs, urinary retention, and fecal incontinence, worsening over a year. During the neurologic examination, she was alert with full orientation but could not walk without assistance, scoring an EDSS of 6.5, mRS of 4, and CASE of 3. Brain MRI revealed T2 high signal intensities in the bilateral subcortical and periventricular white matter, middle cerebellar peduncle, and cerebellum (Fig. 1C). The cerebrospinal fluid (CSF) test showed mild leukocytosis with a high immunoglobulin G (IgG) index (8/μL white blood cells [WBCs], 100% leukocytes, 47 mg/dL protein, and 3.04 IgG index) and oligoclonal band (Type 3) (Table 1). Extensive laboratory evaluations of infection, autoimmune diseases, and malignancy were all negative, and empirical steroid treatment also failed. To exclude the possibility of malignancy, such as gliomatosis cerebri, a brain biopsy was performed, revealing CD68‐positive microglia/macrophage infiltration with a few scattered CD3‐positive T cells (Fig. 1A). She was clinically diagnosed with PPMS for which no treatment is available, and ocrelizumab is not regionally approved. Empirical immunotherapies, including IVIg, rituximab, tocilizumab, and tofacitinib, were ineffective.

Because the pathological findings indicated CAIR, anakinra treatment was sympathetically applied with the patient's consent four and a half years from disease onset. The treatment was very effective. Her symptoms dramatically improved, allowing her to perform daily activities and walk without any assistance, scoring an EDSS of 2, mRS of 2, and CASE of 1. The brain lesions on MRI also disappeared during 5 months of treatment (Fig. 1C). The anakinra treatment continued for 5 months without any side effects. However, after switching from anakinra to azathioprine for maintenance treatment due to cost issues, the clinical symptoms and MRI lesions recurred in 1 month (Fig. 1C), and she subsequently resumed the anakinra treatment.

### Case 2 (Patient 2)

A 51‐year‐old male was transferred to our hospital due to an altered mentality (Fig. 1B and C). He previously suffered from chronic rhinitis with conjunctivitis. His symptoms started with headache and fever, and within a week, he experienced a rapid cognitive decline with memory loss to a point where he could not even recognize his own writing. During the neurologic examination, he was drowsy and disoriented to time and place with severe memory impairment, scoring CASE of 12, mRS of 5, and mini‐mental status exam of 19. The initial CSF test showed leukocytosis with elevated protein (172/μL WBCs, 23% poly, 55% leukocytes, 22% others, and 85 mg/dL protein), and brain MRI revealed T2 lesions in the bilateral medial temporal lobes and basal ganglia with leptomeningeal enhancement (Fig. 1C). Extensive evaluation of infectious etiologies (viral, fungal, bacterial, and mycobacterium), autoantibody‐mediated diseases, and malignancy was negative. Because high‐dose corticosteroids followed by serial immunotherapies, including IVIg, rituximab, and tocilizumab, were ineffective, a brain biopsy was performed, revealing chronic granulomatous and suppurative vasculitis with diffuse infiltration of CD68‐positive macrophages (Fig. 1B).

To target CAIR caused by GPA, we treated the patient with anakinra 10 weeks after disease onset, and the treatment continued for 2 weeks. The patient responded very well to the treatment, showing rapid improvement in symptoms. He recovered a CASE of 0 and mRS of 1 with minimal memory impairment, and the T2 lesions in the mesial temporal lobes also clearly decreased with no more leptomeningeal enhancement (Fig. 1C). He returned to daily life without recurrence while taking oral prednisolone and azathioprine maintenance.

## Discussion

The off‐label use of anakinra in cerebral autoinflammatory–autoimmune diseases showed therapeutic potential in four patients who had a poor response to conventional immunotherapy. In particular, three patients with pathological findings of CAIR were clearly responsive to anakinra, and these patients were clinically diagnosed with PPMS, GPA, and ADEM. Therefore, these findings suggest that anakinra may be the primary treatment for a certain group of cerebral autoinflammatory–autoimmune diseases.

Anakinra controls dysregulated innate immunity by blocking IL‐1‐mediated autoinflammatory reactions.^11^ Systemically, myeloid cells play a crucial role in generating autoinflammasomes by recognizing pathogen‐associated molecular patterns or damage‐associated molecular patterns and potentiating the autoinflammatory response by secreting IL‐1 and IL‐18.^8^, ^15^ In our patients with CAIR, while the exact pathogenesis of activated microglia or macrophage infiltration across the blood–brain barrier remains unclear, the secretion of IL‐1 from microglia or macrophages may have maintained CAIR through a vicious cycle. In Patient 1, who was clinically diagnosed with PPMS, the autoinflammatory response may have originated from peripherally infiltrated macrophages and activated microglia. Indeed, a high concentration of blood monocyte‐driven IL‐1β has been associated with a poor prognosis of PPMS.^16^ Patient 2, who was diagnosed with GPA, showed a very good response to a short‐term treatment with anakinra. As discussed in the previous case report of Patient 3,^7^ persistent microglial dysregulation may have occurred, resulting in CAIR in Patient 2. Although we could not confirm the pathology of Patient 4, the pathogenesis of ADEM may be similar to that of Patient 3 according to the response to the anakinra treatment.^7^ In Patients 2 and 4, the seizures were clearly improved by anakinra, and the inhibition of IL‐1 may be critical for normalizing their seizure susceptibility.^17^ However, some patients did not respond to anakinra even if they were clinically diagnosed with ADEM or pathologically showed microglial infiltration. These results suggest that even with the same diagnosis, the pathogenesis may differ. Thus, nonfoamy microglia in the pathology of Patients 8 and 9 may not play a major role in the pathogenesis. Moreover, although microglial activation has been reported in methotrexate‐necrotizing leukoencephalopathy, JBE, and CJD,^18^, ^19^, ^20^ the inhibition of IL‐1 was ineffective in our patients, and dysregulated innate immunity may not be the main force driving pathogenesis. The CSF cytokine profile could be helpful in the diagnosis of CAIR, but according to previous studies,^7^, ^13^, ^21^ it could not be easily detectable due to the low threshold even if released into CSF, and IL‐1β might be locally active in inflammatory sites. Therefore, it could be valuable to measure local CSF or perform single‐cell RNA sequencing of locally infiltrated microglia in the future.

The present study demonstrates the presence of CAIR in a selected population of patients with refractory cerebral autoinflammatory–autoimmune diseases, and anakinra was effective in these patients. Although this study was a retrospective study with a small number of patients, our pilot data with the anakinra treatment may provide a useful option for refractory cerebral autoinflammatory–autoimmune diseases. Further research is required to determine CAIR in patients with refractory cerebral autoinflammatory–autoimmune diseases and analyze the optimal candidates for anakinra treatment.

## Ethical Approval and Consent to Participate

We confirm that we read the journal's position regarding issues involved in ethical publication and affirm that this report is consistent with these guidelines. This study was approved by the Seoul National University Hospital Institutional Review Board.

## Consent for Publication

Written informed consent was received from all patients and/or legal guardians.

## Availability of Data and Materials

The datasets used and/or analyzed during the current study are available from the corresponding author upon reasonable request.

## Conflict of Interests

The authors declare that they have no competing interests.
